# Financial evaluations of antibiotic stewardship programs—a systematic review

**DOI:** 10.3389/fmicb.2015.00317

**Published:** 2015-04-16

**Authors:** Jan-Willem H. Dik, Pepijn Vemer, Alex W. Friedrich, Ron Hendrix, Jerome R. Lo-Ten-Foe, Bhanu Sinha, Maarten J. Postma

**Affiliations:** ^1^Department of Medical Microbiology, University of Groningen, University Medical Center GroningenGroningen, Netherlands; ^2^Unit of PharmacoEpidemiology & PharmacoEconomics, Department of Pharmacy, University of GroningenGroningen, Netherlands; ^3^Department of Epidemiology, Institute of Science in Healthy Aging & health caRE (SHARE), University Medical Center GroningenGroningen, Netherlands; ^4^Certe Laboratory for Infectious DiseasesGroningen, Netherlands

**Keywords:** antibiotic stewardship, antibiotic resistance, health economics and outcomes research, costs and costs analysis, economic evaluation

## Abstract

**Introduction:** There is an increasing awareness to counteract problems due to incorrect antimicrobial use. Interventions that are implemented are often part of an Antimicrobial Stewardship Program (ASPs). Studies publishing results from these interventions are increasing, including reports on the economical effects of ASPs. This review will look at the economical sections of these studies and the methods that were used.

**Methods:** A systematic review was performed of articles found in the PubMed and EMBASE databases published from 2000 until November 2014. Included studies found were scored for various aspects and the quality of the papers was assessed following an appropriate check list (CHEC criteria list).

**Results:** 1233 studies were found, of which 149 were read completely. Ninety-nine were included in the final review. Of these studies, 57 only mentioned the costs associated with the antimicrobial medication. Others also included operational costs (*n* = 23), costs for hospital stay (*n* = 18), and/or other costs (*n* = 19). Nine studies were further assessed for their quality. These studies scored between 2 and 14 out of a potential total score of 19.

**Conclusions:** This review gives an extensive overview of the current financial evaluation of ASPs and the quality of these economical studies. We show that there is still major potential to improve financial evaluations of ASPs. Studies do not use similar nor consistent methods or outcome measures, making it impossible draw sound conclusions and compare different studies. Finally, we make some recommendations for the future.

## Introduction

The therapeutic use of antimicrobials in clinical medicine is continuing to be suboptimal. Both overtreatment, with regard to spectrum and duration, and suboptimal treatment, with regard to dosage and most effective therapy, are areas of concern. Either one can lead to an increase in the resistance of bacteria (Goossens, 2009), and unnecessary side effects, including a potentially large economic burden (Gandra et al., 2014). It is thus imperative that action is taken (Carlet et al., 2011; World Health Organization, 2012). Fortunately, many hospitals are aware of this, and act accordingly by implementing Antimicrobial Stewardship Programs (ASPs) in their institutions. These programs differ in approach, but the consensus is that when implemented correctly, considerable positive (clinical) effects can be attained by optimizing patients' antimicrobial therapy (Davey et al., 2013). Notably, problems such as increased antimicrobial resistance, consequent treatment failure, and the spread of nosocomial infections can be prevented with ASPs, inclusive its financial consequences (Roberts et al., 2009). Implementing an ASP can thus also have a considerable positive financial impact within a hospital, which is crucial in times where healthcare costs are rising.

There are various guidelines published describing to design an ASP (Dellit et al., 2007; SWAB, 2012; With de et al., 2013), consisting of a set of interventions and services, all with the goal to stimulate the correct use of antimicrobials, but intervening at different moments in the chain of care. One or more ASPs can be implemented, depending on the type of hospital, the types of patients and the local challenges that physicians face. In general, all tasks within an ASP can be categorized within three blocks:

- The first block consists of tasks that can be performed during the start of empirical antimicrobial therapy (the so-called “front-end” approach). Examples are pre-analytic consultations and providing therapy guidelines and education for prescribing doctors.
- A second block consists of tasks to assist by the optimization of the therapy around day 2–3, such as interventions to promote IV to oral switch, de-escalation and a timely stop when appropriate (the “back-end” approach).
- Finally, a last block of supplemental tasks should assure evaluation of hospital data and tasks that act upon those data accordingly, like updating guidelines using local resistance rates and processes, as well as promoting surveillance studies (Dellit et al., 2007; SWAB, 2012; With de et al., 2013).

Whether the intervention is restrictive or persuasive does not seem to differ on the long-term—i.e., after 6 months of implementation (Davey et al., 2013).

During the last years, there is a steady rise in papers published on above mentioned interventions. Coinciding with that rise, the number of economic evaluations of an ASP is also increasing. This reflects the growing importance of health economic evaluations in general, which is seen across the world, as well as that of ASPs in particular (Hjelmgren et al., 2001). However, there seems to be a large variation in the way ASPs are financially evaluated. Often only the direct costs for antimicrobials is taken into account, whereas other (in)direct costs may have a much larger impact (McGowan, 2012; Davey et al., 2013). Many papers mention some costs and/or benefits, but only a few give usable, in-depth data, analyses and conclusions. When comparing financial ASP results, for example, within the latest Cochrane review, the conclusion was that economic evaluations are done in a “disappointingly” low number of studies and often lack reliable data (Davey et al., 2013).

This study provides a systematic methodological review of published economic evaluations of ASP studies (intervention studies with an economic evaluation paragraph or complete economic evaluations). Ideally, it will shed light on the divergence that is present in these studies and where improvement is necessary. Keeping in mind that decision makers use these economic evaluations in their daily practice nowadays, and costs are considered a barrier to implement an ASP, correct and valuable studies are becoming more important (Johannsson et al., 2011). This review will therefore in particular look at the methods' usability for others that might want to implement an ASP in their hospital.

## Materials and methods

**Figure 1:**
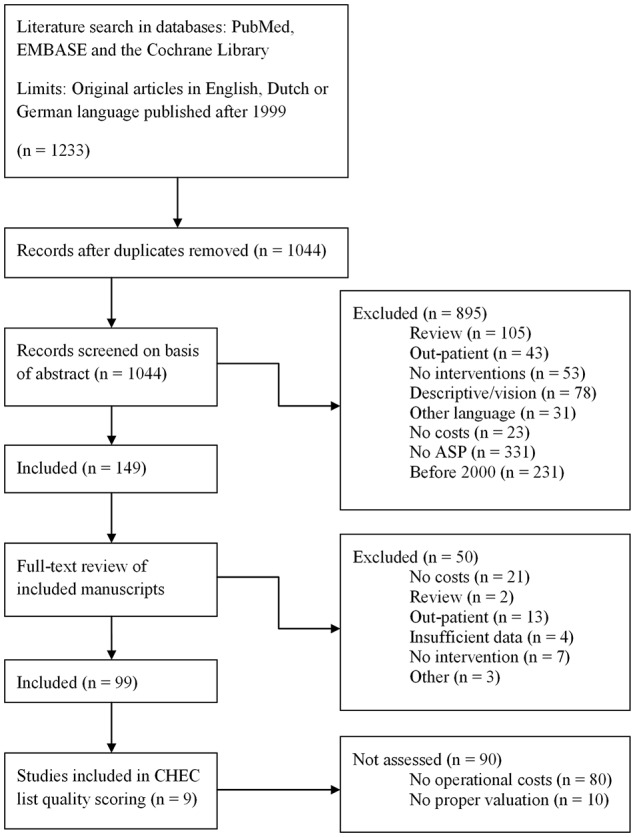
**Flow chart of the search method**. The followed search method as performed on 4 November 2014.

A search was performed within the PubMed and EMBASE databases in November 2014, using the following search strings: “antimicrobial stewardship,” “antimicrobial management,” “antimicrobial prescribing intervention,” and “antimicrobial program intervention.” All strings were in combination with the words “cost(s),” “financial,” “economic,” “dollar” or “euro,” or the respective symbols for the latter two. All abstracts found were read and original studies written in English, Dutch or German that discussed an intervention the related economic analysis of that intervention within a hospital were included. Considering the fast developments within the field of antimicrobial stewardship as well as within health economics, studies before 2000 were excluded. Outpatient settings were excluded (see Figure 1 for the complete flow chart).

The final set of included papers was read completely, and for each study the type of economic evaluation done was scored. Keeping in mind that many studies are clinical effect studies and not economic analyses studies, the papers that only mentioned direct antimicrobial costs without mentioning any of the other relevant costs or savings, were categorized as an Antimicrobial Cost Analysis (ACA). The remainder of the studies contained enough essential financial parameters to be classified as different economic analyses (e.g., as described in Drummond et al., 2005). For studies looking at the effects of two methods and that converted those effects into monetary values the classification Cost-Benefit Analyses (CBA) was used. Those that evaluated the relative costs and effects of two different methods were classified as Cost-Effectiveness Analyses (CEA). Studies observing the different costs and effects of two methods were classified as Cost-Consequence Analyses (CCA). Studies that looked at the costs but not at the effects were scored as Cost-Analyses (CA) and studies that looked at the differences in costs assuming similar effects between the two evaluated methods were scored Cost-Minimization Analyses (CMA). For the papers the following parameters were scored: year, journal, country of research, study design, setting, number of participants, outcome measures, price adjustment, and/or discounting measures, and conclusions. The types of intervention per study were scored and categorized.

Within an economic analysis, different costs can be taken into consideration. Drummond et al. recommend calculating operational costs and capital costs that are related to the intervention (Drummond et al., 2005). To further specify this for an ASP intervention study, the outcome measures from the Cochrane review were taken as specific parameters (Davey et al., 2013). Depending on the perspective chosen, the following parameters were scored: implementation costs, operational costs (personnel and/or equipment costs of the intervention), antimicrobial costs, hospital day costs, morbidity, and/or mortality costs (costs associated with hospital procedures, treatment etc.), societal costs (costs occurring outside the hospital from a societal perspective, e.g., loss of productivity), and other costs (costs mentioned by a study that are different than already mentioned here).

In order to assess the level of quality of the included papers, an appropriate quality criteria list (Consensus on Health Economics Criteria [CHEC] list) was used (Evers et al., 2005). A criteria list specifically intended to give insight in the quality of economic evaluations. Considering the fact that many studies lacked a proper economic analysis it was not deemed of great interest or informative doing an in depth quality assessment on all papers of which the majority would not meet minimum criteria. We therefore decided to only formally assess the quality in detail if the paper met minimum standards. Two parameters were assumed as most basic and essential for an economic evaluation studies and used as an inclusion criterion: implementation costs and/or operational costs and appropriate valuation of all costs. Articles that included these parameters were consequently scored following the CHEC list by two authors independently.

This review study followed the PRISMA criteria where possible (Moher et al., 2009). The complete checklist can be found as Supplementary Material (Supplemental Table 1).

## Results

For this review, a total of 1233 papers were found using our search strings. Of these papers, 1083 were excluded based upon the fact that they did not meet our inclusion criteria (for example, because they were review papers, there were no cost outcome measures mentioned or they were published in a non-included language). One hundred and forty-nine papers were included and read completely. Of these papers, a further 50 were excluded for various reasons. A set of 99 papers was included in the final analysis (Figure 1).

### Baseline characteristics

From the total of 99 papers, the majority came from the United States followed by Europe and most included studies were published within the last 3 years. Studies were performed in hospitals ranging from as little as 39 beds to as much as 1800 beds and included between 50 and 40,000 patients. For a complete overview see Table 1.

**Table 1:** **General characteristics of the reviewed studies (*n* = 99)**.

**Characteristic**	**Number**	**Percentage**
**GEOGRAPHY**		
North America	51	52%
South America	3	3%
Europe	28	28%
Asia	14	14%
Africa	2	2%
Australia	1	1%
**PUBLICATION YEAR**		
2000–2003	8	8%
2003–2006	14	14%
2006–2009	17	17%
2009–2012	13	13%
2012–2014	47	47%
**STUDY DESIGN**		
ITS	8	8%
Quasi-experimental study	65	66%
Retrospective evaluation	12	12%
(R)CT	8	8%
Cost-analysis	2	2%
Observational study	3	3%
Unclear	1	1%
**NUMBER OF BEDS IN HOSPITAL**		
<150	10	10%
150–500	24	24%
500–1000	27	27%
>1000	12	12%
Unclear	23	23%
**NUMBER OF PATIENTS INCLUDED**		
<100	10	10%
100–250	22	22%
250–500	14	14%
500–1000	6	6%
1000–1500	6	6%
>1500	10	10%
Unclear	31	31%

### Types of interventions

Categorizing the stewardship interventions in block 1 (front-end approach), block 2 (back-end approach), and block 3 (supplemental measures), most studies implemented one or more interventions from block 2 (Table 2). Particularly, the implementation of an audit of and/or feedback on the therapy provided at certain time-point(s) was performed frequently (62 studies; 52% of all interventions). Second most frequent was the creation and implementation of antimicrobial therapy guidelines (16 studies, 13% of all interventions). 18 studies (18% of all studies) implemented more than one intervention at the same time, providing a “bundle” of services.

**Table 2:** **Types of interventions of the reviewed papers**.

**Block**	**Interventions**	**Number**	**Percentage**	**Patients**
1	Altered therapy guidelines	16	16%	32,103
	Antibiotic restriction lists or pre-authorization	12	12%	70,446
	Giving education	10	10%	21,913
	Antibiotic cycling	1	1%	–
	Pre-analytic consultations	1	1%	100
	New therapy	1	1%	2888
2	Therapy evaluation, review and/or feedback	62	63%	51,506
	Rapid diagnostic tools	9	9%	701
	New biomarkers	2	2%	–
3	Producing local use and resistance data	5	5%	19,390

Performed interventions per category, the number of studies that evaluated this intervention, the percentage of the total and the total number of patients (if this was mentioned in the papers).

### Types of analyses

Fifty-seven (58%) papers only included antimicrobial costs as an economic outcome measure without any of the other relevant costs. Although these studies looked at costs and effects, they could not be classified as a proper economic analysis, because an appropriate economic evaluation was not done. They were therefore classified as an ACA. Of the rest, the majority, 32 were scored as a CBA. Three studies evaluated the relative costs and effects classifying them as a CEA. There were 3 CCAs, 2 studies only looked at the costs making it a CA and 2 studies were CMAs (Table 3).

**Table 3:** **Types of economic evaluation of the reviewed papers**.

**Type of analysis**	**Number**	**Percentage**
CA	2	2%
CMA	2	2%
CBA	32	32%
CCA	3	3%
CEA	3	3%
CUA	0	0%
ACA	57	57%

CA, Cost-Analysis; CMA, Cost-Minimization Analysis; CBA, Cost-Benefit Analysis; CCA, Cost-Consequence Analysis; CEA, Cost-Effectiveness Analysis; CUA, Cost-Utility Analysis; ACA, Antimicrobial Cost Analysis.

### Cost outcome measures

For every study, the cost outcome measures they used were scored. None of the papers included all. Every study took a hospital perspective when looking at the costs and benefits, although only a few explicitly mention this. None of the studies performed their analysis from a societal perspective, although comments were made on the importance of including these costs. Disregarding the societal costs, there were 3 (3%) studies that included all of the other parameters (Frighetto et al., 2000; Gross et al., 2001; Hamblin et al., 2012), 3 (4%) more studies included all but implementation costs (and, as stated, societal costs) (Bauer et al., 2010; Niwa et al., 2012; Perez et al., 2013). Fifty-seven studies (58%) only included antimicrobial costs (Table 4).

**Table 4:** **Scored outcome parameters of the reviewed papers**.

**Outcome measures**	**Number**	**Percentage**
Implementation costs	11	11%
Antimicrobial costs	97	98%
Operational costs	23	23%
LOS costs	18	18%
Morbidity/mortality costs	14	14%
Other hospital costs	19	19%
Societal costs	0	0%

LOS, length of stay.

### Quality assessment

Following the criterion as stated in the Material and Methods section, 9 studies included operational costs of the intervention and appropriately valued their costs by taking into account inflation and/or price changes (Al Eidan et al., 2000; Frighetto et al., 2000; Gross et al., 2001; Ansari et al., 2003; Rüttimann et al., 2004; Oosterheert et al., 2005; Hamblin et al., 2012; Sick et al., 2013). For these studies, quality was assessed following the CHEC list and results are mentioned in Table 5. Results ranged between 2 and 14 positives on the criteria of a maximum of 19, with 3 studies scoring less than 10. An incremental analysis of the costs and health outcomes, and sensitivity analyses were among the items most frequently missed.

**Table 5:**
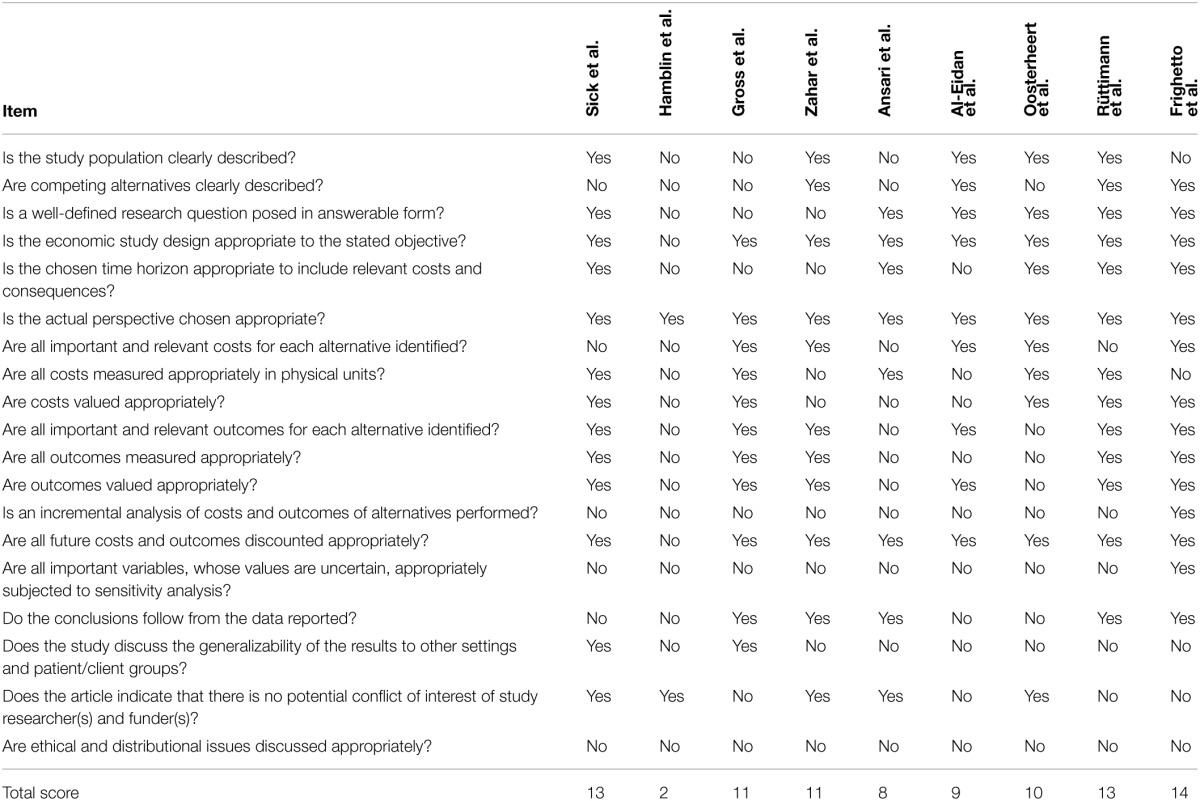
**Results of CHEC list score**.

## Discussion

Concluding it can be said that economic evaluations of ASPs have room for improvement. Often the methods chosen are insufficient to be conclusive, because essential parameters are missing and multiple different approaches to evaluate an ASP are being used. It is therefore in most cases difficult if not impossible to translate results to other settings. Even relatively simple parameters, such as number of patients, study design and setting, perspective chosen, performed statistics, and inflation corrections are frequently missing. Often the evaluation was done on a set of interventions, making their respective benefits undistinguishable. The lack of quality is further reflected in the fact that only 9 studies could be included in the quality assessment and none scored above 14 (of a maximum of 19), underlining the need for a more systematic approach for these types of studies. A meta-analysis of published results, which had the preference, could therefore not be performed. Thus, the focus of this review was switched to highlighting the possible areas for improvement.

The primary goal for an ASP should be to improve patient outcomes and quality of care. It is shown that running an effective ASP can optimize antimicrobial therapies thereby improving patients' treatment (Davey et al., 2013). This can in turn positively affect local resistance rates and local nosocomial infections rates. Some studies also showed the positive effect an ASP could have on the length of stay (LOS) of a patient (e.g., Al Eidan et al., 2000; Frighetto et al., 2000; Niwa et al., 2012; Yen et al., 2012; Perez et al., 2013; Rimawi et al., 2013; Sick et al., 2013). Such a reduction can improve patient safety and quality of care as well. Keeping this in mind, it is worthwhile to also look at the effectiveness of stewardship programs, especially from an economical point of view. Obviously, it is highly preferably if this is performed according to a set of guidelines (Drummond et al., 2005). We have shown that major room for improvement in this area still exists.

Ideally, an economic evaluation begins with defining the type of intervention that is performed and the perspective that is chosen to do the evaluation. Most papers reviewed seemingly chose a hospital perspective, although few mention this explicitly. A societal perspective might be preferred in many cases, since patients and society in general will benefit from the intervention, especially when a reduction in the antimicrobial resistance rates is achieved, or when LOS is reduced. By merely looking at it from a hospital perspective, such benefits are lost or marginalized (Chen, 2004). Effects of interventions are dependent on the local epidemiological data on resistance, and consequently this will influence the total benefits and should thus not be overlooked.

If the economic evaluation is done from a hospital perspective, the costs of the program will consequently be the ones made by the hospital. These can be categorized as fixed and variable. Fixed costs are those that do not vary with the quantity of output (i.e., number of patients) in the short run and include for example rent, equipment, maintenance etc. (Drummond et al., 2005). For hospitals, studies showed that fixed costs can range from 65% up to 84% of the total budget. The latter percentage also included the personnel costs in their calculations (Roberts et al., 1999; Taheri et al., 2000). Staff salaries can be considered fixed or semi-fixed, especially on the short-term. This means that for an ASP, which is often evaluated on the short-term, direct cost reductions in practice can primarily be expected in the variable costs. There is a need for a longer term perspective in economic evaluations of ASPs.

Almost all interventions require time, resources, and sometimes equipment to implement. These costs should not be overlooked. Not all community hospitals have the same resources available as large academic centers, and implementation costs can therefore be a major hurdle (Johannsson et al., 2011). Of the reviewed papers however, only 11 studies (11%) mentioned the costs spent on implementation.

For a majority of the reviewed studies (58%), the only included cost outcome parameter was the cost price of antimicrobials. These costs are obviously easier to obtain and measure than others. It is also one of the only ways for an ASP to significantly influence the variable budget of the hospital. When evaluating the costs of antimicrobials however, it is still important to adjust prices to a single year/level in order to remove the bias of changing prices/inflation. Of the included studies only 14 (14%) did so. Prices of antimicrobials change over time, due to inflation, but also because patents can expire and (cheaper) generic substitutes can become available. This becomes especially important when a study evaluates several years of antimicrobial acquisition costs. The longer the study period is, the higher the chance that more generic substitutes became available due to expired patents. With more than 5 generic products entering the market, prices of the original brand antibiotic can drop by more than 75% (FDA, 2010). Furthermore, prices yearly chance in reality due to changing agreements between hospitals and external parties. It is therefore essential that during a financial evaluation of an ASP the prices are fixed for the whole study.

Performing the interventions, costs time and time spent invokes opportunity costs. This implies that the time doctors or pharmacists spend on the ASP cannot be spent on something else. This time should thus also be included in an economic evaluation. There are multiple ways to measure the time that was put into the program and each method has his pros and cons, as indicated by Page et al. (2013) in an overview of various methods (Page et al., 2013). When looking at the total costs of the time spent, it is important to include all personnel costs (salary and all related attributable on-costs) to give the most realistic monetary output.

One of the results of an ASP can be the reduction of LOS. From a financial point of view such a reduction is highly interesting because of the high costs that are associated with it. Costs for a hospital day are however almost completely fixed and will therefore not change over time due to fewer patient hospital days, unless wards or beds are closed, but even then depreciation costs are made (Rauh et al., 2010). This is something to take into consideration. An important question is thus how to value a possible reduction of the LOS. For hospital days there is no market to establish a price (Scott et al., 2001). This makes it difficult to aptly include the effect on LOS in an economic evaluation. Often, studies look at the costs (fixed and variable) made at a department or hospital and divide this by the patient days. An indication for these costs can be calculated by accounting all costs; by looking at the incremental costs for the last (cheaper) day(s) (Taheri et al., 2000); or by willingness to pay (Stewardson et al., 2014). The latter study gave a fascinatingly low figure compared to the other methods, showing that high prices for a hospital day are not always realistic depending on the perspective. Of the reviewed papers that mentioned their cost for a hospital day, the average in 2013 Euro level, was €649.04 per hospital day (Al Eidan et al., 2000; Barenfanger et al., 2000; Frighetto et al., 2000; Oosterheert et al., 2005; Forrest et al., 2006; Niwa et al., 2012; Yen et al., 2012; Maddox et al., 2014). However, because it was not always clear which costs were included in this figure, it is almost impossible to draw conclusions based on these numbers. A reduction in LOS can thus have a difficult to estimate effect on the balance. Freeing up beds does have however an important positive benefit in the form of an increased possibility to boost the hospital's turnover. Reduction of hospital days is thus especially interesting if the backfill of a hospital is large enough to fill up the freed beds. If this is not the case, fixed costs are divided over less patient days, and depending on the cost structure, this can even mean hospital costs rise per patient. Additionally, from a patient perspective there are huge benefits in leaving a hospital earlier. These are covered by taken a broader perspective in the analysis.

A subsequent broader perspective for economical evaluations is looking at an intervention from a health payer perspective. This entails the inclusion of costs made by the patient, such as home medication and costs for a general practitioner. For a societal perspective, additional indirect and non-medical costs that occur outside the hospital are included, such as expenses made by patients like transportation and spent time, but also loss of productivity for the society (Drummond et al., 2005). Furthermore, a patient can live longer or with better quality of life due to an intervention. One method to take these effects into account is to measure the quality adjusted live years (QALYs) in a long-term analytic approach. Together with the costs of the intervention, a cost-effectiveness ratio can then be calculated. Depending on the threshold for a QALY gained, an intervention can be judged financially worthwhile to implement or not. The inherent difficulties of this method (e.g., time and knowledge needed to perform this extensive evaluation), especially for a relatively small intervention program as an ASP, are illustrated by the fact that no study performed such an analysis. In an outpatient setting however, Oppong et al. (2013), is a nice example of such a study that did this analysis.

Results of an economic evaluation of an ASP can be used to convince a board of directors that pro-actively spending money on improving antimicrobial therapies can give a positive return of investment (Johannsson et al., 2011). However, when this is the main goal of the evaluation, a simple cost (benefit) analysis is not enough, because it will miss effects outside the hospital. Cost effectiveness/utility is more precise, but complex. Preferably, the department or the hospital therefore makes a business case model for an ASP in order to give an, as complete as possible financial overview. Both Stevenson et al. (2012) and Perencevich et al. (2007) proposed a similar set-up for this.

To assess the quality of the included papers within this review, the CHEC list was used (Evers et al., 2005). The Consolidated Health Economic Evaluation Reporting Standards (CHEERS) Statement has been used to evaluate the quality of CEAs in reviews (Husereau et al., 2013). See for example Abu Dabrh et al. (2014), Hop et al. (2014), and Kawai et al. (2014). However, in our opinion, the CHEERS checklist is not suitable for this specific review. It only considers whether something is reported, not whether the choices were appropriate or justified. As such, a checked box on the CHEERS checklist is not an assessment of quality, merely one of completeness. Another recent publication, the checklist by Caro et al. was specifically designed for a reliability and credibility assessment. However, this checklist is only applicable for decision models, and as such was not appropriate for our review (Caro et al., 2012). Therefore, the CHEC list was chosen, while this, as said, specifically gives insight in the quality of economic evaluations (Evers et al., 2005).

Concluding, it can be said that there is still much room to improve economic evaluations of ASPs. Of the papers reviewed, none can really be used to draw strong economical conclusions. Using more standardized methods to financially evaluate an ASP will contribute to the advancement needed in this field and notably, further research should focus on the harmonization of this field. Finally, inclusion of the societal perspective, real-world pricing and a potentially longer time horizon for analysis can be recommended. Ultimately, these improvements should provide a more solid basis for decision making, potentially leading to better patient care.

## Author contributions

JD, AF, MP, and BS contributed to the initial conception and the design of the study; JD performed the database searches and analyses; JD and PV performed the quality assessment; JD, JL, and BS drafted the manuscript and all authors revised the work critically and gave final approval before publication.

### Conflict of interest statement

BS has received a travel grant co-funded by Pfizer/Wyeth, and worked on projects in cooperation with Pathogenica Life Technologies, and Copan. The other authors declare that the research was conducted in the absence of any commercial or financial relationships that could be construed as a potential conflict of interest.
